# A comparison between virus- versus patients-centred therapeutic attempts to reduce COVID-19 mortality

**DOI:** 10.1080/22221751.2021.2006579

**Published:** 2021-12-01

**Authors:** Serge Camelo, Mathilde Latil, Sam Agus, Waly Dioh, Stanislas Veillet, René Lafont, Pierre J. Dilda

**Affiliations:** a Biophytis, Sorbonne Université, Paris, France; b Biophytis Inc., Cambridge, MA, USA; c CNRS – Institut de Biologie Paris Seine (BIOSIPE), Sorbonne Université, Paris, France

**Keywords:** ACE2, aging, antivirals, COVID-19, dexamethasone, mas receptor, renin-angiotensin system, Tocilizumab

## Abstract

Since December 2019, coronavirus disease 2019 (COVID-19), caused by infection with severe acute respiratory syndrome coronavirus 2 (SARS-CoV-2), has changed our lives. Elderly and those with comorbidities represent the vast majority of patients hospitalized with severe COVID-19 symptoms, including acute respiratory disease syndrome and cardiac dysfunction. Despite a huge effort of the scientific community, improved treatment modalities limiting the severity and mortality of hospitalized COVID-19 patients are still required. Here, we compare the effectiveness of virus- and patients-centred strategies to reduce COVID-19 mortality. We also discuss the therapeutic options that might further reduce death rates associated with the disease in the future. Unexpectedly, extensive review of the literature suggests that SARS-CoV-2 viral load seems to be associated neither with the severity of symptoms nor with mortality of hospitalized patients with COVID-19. This may explain why, so far, virus-centred strategies using antivirals aiming to inhibit the viral replicative machinery have failed to reduce COVID-19 mortality in patients with respiratory failure. By contrast, anti-inflammatory treatments without antiviral capacities but centred on patients, such as dexamethasone or Tocilizumab^®^, reduce COVID-19 mortality. Finally, since the spike protein of SARS-CoV-2 binds to angiotensin converting enzyme 2 and inhibits its function, we explore the different treatment options focussing on rebalancing the renin-angiotensin system. This new therapeutic strategy could hopefully further reduce the severity of respiratory failure and limit COVID-19 mortality in elderly patients.

## Introduction

Since the outbreak of the coronavirus disease 2019 (COVID-19) pandemic [1], a tremendous effort of the scientific community has taken place to explore any therapeutic option able to reduce COVID-19 death toll. Massive vaccination campaigns aiming to limit the spread of the severe acute respiratory syndrome coronavirus 2 (SARS-CoV-2) are ongoing [2]. In parallel, the management of critically ill patients has been continuously improved, thanks to oxygen therapy and antithrombotic agents. However, despite these efforts, COVID-19 mortality that particularly affects the elderly population often displaying underlying comorbidities [3] remains high. Thus, in the context of emerging more infectious SARS-CoV-2 variants [4], new therapeutic options limiting the severity and mortality of hospitalized COVID-19 patients are still required.

It has been proposed that high viral load and excessive inflammation are associated with acute respiratory disease syndrome (ARDS), cardiac dysfunction, renal alterations and mortality following SARS-CoV-2 infection [3,5]. Treatment options can be classified depending on whether they aim to block the virus entry into cells or its replication in order to reduce SARS-CoV-2 viral load, or whether they intend to limit the clinical consequences of infection for the patients. Here we review what treatments have been tested, and we compare the effectiveness of virus- versus patients-centred therapeutic strategies to reduce COVID-19 mortality. We also discuss the potential role of the renin-angiotensin system (RAS) imbalance in the increased susceptibility of older people to COVID-19 mortality.

## Materials and methods

We performed an extensive review of the literature published on COVID-19 between January 2020 and August 2021. Although not an exhaustive review of the literature, relevant articles were identified through searches in the authors’ personal files, in PubMed and Research Gate for articles published in English by use of the terms “COVID-19” or “SARS-CoV-2,” and “Remdesivir,” “Hydroxychloroquine (HCQ),” “Chloroquine,” “Lopinavir,” “Ritonavir,” “Favipiravir,” “Molnupiravir,” “viral load,” “viral shedding,” “convalescent plasma (CP),” “Neutralizing antibodies,” “Tocilizumab,” “Dexamethasone,” “cytokine storm,” “interleukin-6 (IL-6),” “IL-1,” “C-reactive protein (CRP),” “inflammation,” “angiotensin converting enzyme-2 (ACE2),” “Angiotensin-II (Ang-II),” “Angiotensin-(1–7),” “Mas receptor,” “RAS” and “aging.”

### Virus-centred strategies

#### Preventive treatment options in non-hospitalized and patients with mild COVID-19 symptoms

In 2003, the death toll due to infection by the SARS-CoV was limited by careful isolation of patients and by treatments with antiviral drugs [6]. In January 2020, SARS-CoV-2, the virus causing COVID-19, was identified [1]. It was then logical to explore a similar virus-centred therapeutic strategy against COVID-19. Therefore, the use of antivirals to prevent aggravation of the clinical status of patients with mild symptoms or to limit the propagation of the virus in the population was tested. It was rapidly demonstrated that chloroquine, by inhibiting endosome maturation [7], impaired SARS-CoV-2 replication *in vitro* [7,8]. Based on these *in vitro* experimental data, repurposing of chloroquine (and its derivative HCQ, commercialized under the trade name Plaquenil^®^) appeared as an ideal cure against COVID-19. It has been reported that HCQ alone or in combination with azithromycin reduces viral load and accelerates the recovery of outpatients with mild symptoms of COVID-19, but no effect on mortality could be observed [9]. Remdesivir (Veklury^®^) also blocks SARS-CoV-2 replication *in vitro* by inhibiting the viral RNA-dependent RNA polymerase [7,8]. When tested clinically, Remdesivir reduced the time to recovery when applied early in the course of infection in patients with oxygen therapy [10]. Lopinavir^®^/Ritonavir (Kaletra^®^), a combination of putative inhibitors of the main SARS-CoV-2 protease, reduced SARS-CoV-2 replication *in vitro* [7]. However, treatments with Kaletra alone or in combination with Favipiravir could not provide clinical benefit in management of mild COVID-19 patients [11]. Ivermectin was shown to limit SARS-CoV-2 nuclear import and thus its replication *in vitro* [12]. However, Ivermectin did not promote any clinical beneficial effects in COVID-19 infected persons with mild symptoms as well [13]. Due to the fact that mortality in this patient’s population is (fortunately) rare, and because some of these trials were underpowered, no definitive conclusion on the effectiveness of early treatment with these antiviral drugs could be drawn from these studies [14]. Recently, however, early treatment with a new antiviral compound, Molnupiravir (MK-4482/EIDD-2801), a pro-drug of the nucleoside analogue *N*^4^-hydroxycytidine, which induces errors during virus replication, has been tested in a large clinical trial in non-hospitalized (NCT04575597) COVID-19 patients. On the first of October 2021, Merck announced in a press release that Molnupiravir reduces the rate of hospitalization by 50% and diminishes the risk of deaths related to COVID-19. Based on these results, a request for an emergency use authorization (EUA) for Molnupiravir to treat non-hospitalized patients at risk is ongoing.

Supplementation of patient’s immunity with neutralizing antibodies or CP has also been proposed to treat COVID-19 patients. SARS-CoV-2’s spike protein specific neutralizing antibodies or CP aim to prevent SARS-CoV-2 entry into cells by impeaching virus attachment to its receptor ACE2 [15]. When used as a “preventive” treatment in non-hospitalized “at risk” patients aged 65 years or older or with a Body Mass Index (BMI) of 35 or more, or seronegative, which are at high risk to develop severe symptoms of COVID-19, infusion of CP or cocktails of recombinant neutralizing anti-SARS-CoV-2 antibodies (Bamlanivimab with Etesevimab and Casirivimab with Imdevimab (REGN-COV2)) led to a faster decline of the viral load, an overall reduction of the severity of symptoms and a lower percentage of patients needing hospitalization or a visit to an emergency department (Table 1) [16–18]. Based on these studies, EUAs of neutralizing antibodies cocktails were granted by the US Food and Drug Administration and the European regulatory authorities for the treatment of recently diagnosed patients with mild to moderate COVID-19, and of high-risk patients with a weak immune system.

**Table 1.** Virus-centred therapeutic strategies against COVID-19.

Treatment	Effect on viral load	Effect on inflammation (CRP)	Effect on severity/mortality
*Antiviral drugs*			
HCQ alone or with Azithromycin^a^	↘↘↘	N.T. (potential effect)	None
HCQ alone or with Azithromycin^b^	↘↘↘	N.T. (potential effect)	Shortens duration of mild symptoms
Remdesivir (Veklury^®^)^a^	↘↘↘	N.T.	None
Remdesivir (Veklury^®^)^b^	↘↘↘	N.T.	Shortens time to recovery
Lopinavir^a^	↘↘↘	N.T.	None
Lopinavir and IFN^a^	↘↘↘	N.T.	None
Lopinavir^®^/Ritonavir (Kaletra^®^) alone or with Favipiravir (Avigan^®^) or Baloxavir Acid^a^	↘↘↘	N.T.	None
IFN^a^	↘↘↘	N.T. (potential effect)	None
Ivermectin^b^	↘↘↘	N.T. (potential effect)	None
Favipiravir^b^	↘↘↘	N.T.	None
Molnupiravir^b^	↘↘↘	N.T.	Reduces hospitalization rates by 50% and reduces mortality
*Blocking viral entry into cells*			
CP^a^,^b^	↘↘	N.T. (potential effect)	None
CP^c^	↘↘	N.T. (potential effect)	↘
Neutralizing antibodies (Bamlanivimab and Etesevimab)^c^ or REGN-COV2 (Casirivimab and Imdevimab)^c^	↘↘	N.T.	↘
Bromhexine^a^	N.T.	↘↘	↘↘

Note: N.T., not tested; CRP, C-reactive protein; CP, convalescent plasma; HCQ, hydroxychloroquine; ↘, low reduction; ↘↘, medium reduction; ↘↘↘, strong reduction.

^a^ Hospitalized patients with severe COVID-19.

^b^ Patients with mild COVID-19.

^c^ High-risk patients aged >65 and/or with BMI ≥35 and/or immunodeficient.

In addition to CP or neutralizing antibodies infusion, inhibition of SARS-CoV-2 entry into cells could also be achieved by injections of soluble human recombinant ACE2 [19]. The effects on mortality of clinical trials testing this therapeutic option are waited for. Inhibiting the spike protein priming, mediated by the cellular serine protease TMPRSS2, is another option to impair virus-cell fusion [15]. Bromhexine an inhibitor of TMPRSS2 has been shown to reduce mortality and complication resulting from COVID-19 in patients with mild symptoms [20]. Nevertheless, the results of this small pilot study still require to be confirmed by clinical trials involving a larger number of patients.

### Failure of virus-centred therapies to reduce COVID-19-associated mortality in hospitalized patients with ARDS

Soon after COVID-19 outbreak, it was shown that, when compared to standardized care in hospitalized patients with respiratory failure, HCQ did not reduce mortality associated with COVID-19 [21], even when it was administered in association with azithromycin [22]. Similarly, Remdesivir did not reduce time to recovery in COVID-19 patients with critical illness requiring high-flow oxygen through mechanical ventilation or extracorporeal membrane oxygenation [10]. Nevertheless, Remdesivir was the first drug approved as a cure against COVID-19 [7]. In addition, Kaletra^©^ alone or in conjunction with other antivirals (Favipiravir (Avigan^®^) or Baloxavir acid) also failed to reduce loss of lives in severely ill COVID-19 patients [23–25]. Finally, in hospitalized patients with severe disease, despite a quicker negative seroconversion, treatment with CP was not associated with improvement of clinical symptoms nor reduction of mortality [26,27].

The ineffectiveness of antiviral drugs limiting SARS-CoV-2 replication and of CP infusion to reduce mortality of hospitalized patients due to COVID-19 is disappointing and may appear puzzling. So far, the biological reasons explaining the failure of virus-centred strategies for hospitalized patients have not been described. It could be proposed that new compounds aiming to limit viral loads would be more successful than those that have been repurposed so far. However, another hypothesis based on differences in viral dynamics between SARS-CoV and SARS-CoV-2 could also explain why antiviral drugs reduced mortality induced by SARS but not by COVID-19 [6]. During the surge of SARS in 2003, it was shown that SARS-CoV viral load was closely associated with SARS symptoms occurrence and mortality [28]. Due to genomic similarities between SARS-CoV and SARS-CoV-2, it seemed logical that in hospitalized patients higher SARS-CoV-2 viral load would be positively associated with the severity of COVID-19 symptoms and COVID-19 mortality. This assertion was supported by several studies [29–33]. However, in these cohorts, critically ill patients more prone to die had higher SARS-CoV-2 titres but were also significantly older (sometimes up to 10 years) and presented more comorbidities than patients with milder symptoms and reduced mortality [29–33]. Thus, from these studies, COVID-19 mortality could be equally attributed to age or comorbidities rather than solely to higher SARS-CoV-2 viral load [29–33]. By contrast, several well-balanced studies showed that viral loads were not statistically different between groups of patients with various degrees of COVID-19 severity and mortality [34,35]. Therefore, by contrast with SARS-CoV, it could be hypothesized that in hospitalized patients infected by SARS-CoV-2, viral titres may not be directly related to COVID-19 mortality.

Importantly, comorbidities such as diabetes, hypertension and cardiovascular diseases [36] and age [36–38] have been consistently associated with the severity and mortality of hospitalized COVID-19 patients. However, association between aging and an increase in viral load remains a subject of debate [34,35]. Indeed, some studies show a week association between age and high viremia [35] but other do not [34]. In any case, a week association between age and viral load could not explain the strong association noted between age and mortality of hospitalized patients with COVID-19 [34,35].

Altogether, so far virus-centred strategies targeting the replicative machinery of SARS-CoV-2 or blocking viral entry into cells have failed to reduce the mortality of patients presenting severe COVID-19 symptoms such as ARDS (Table 1) [39,40]. Nevertheless, antivirals and neutralizing antibodies could still play a role by sparing hospital resources through diminishing the length of hospitalization of COVID-19 patients and as a prophylaxis treatment of non-hospitalized patients.

### Patients-centred strategies

#### Importance of drugs with a broad anti-inflammatory spectrum to treat COVID-19 patients

Alternative therapeutic options aiming to reduce the severe consequences of SARS-CoV-2 infection for hospitalized patients have been explored. As in influenza infection, patients-centred strategies might be more effective to reduce COVID-19 mortality of severely ill patients [41]. A proper immunity is crucial to fight viral infections. But SARS-CoV-2 induces a dysregulation of the immune system, including neutrophilia and lymphocytopenia [42,43]. Accordingly, the presence of neutralizing auto-antibodies against type I interferons (IFNs) and defects in IFNs signalling have been associated with up to 25% of the severe COVID-19 cases [44,45]. In parallel, COVID-19 severity and mortality have also been associated with elevated cytokines production (cytokine storm) concomitant with ARDS [42]. This inflammatory reaction could be triggered by the intracellular recognition of viral dsRNA by TLR7 and TLR8 [46]. Moreover, excessive inflammation observed in severely ill patients could be emphasized in the elderly population by immune-senescence [47,48]. Therefore, early in the COVID-19 pandemic, limiting inflammation appeared important in the fight against the disease. Overexpression of IL-6 has been associated with the severity of symptoms in patients infected with SARS-CoV-2 [36–38]. Accordingly, treatment with Tocilizumab^©^ improved survival and recovery time of intensive care COVID-19 patients [49]. Interestingly, effectiveness of anti-IL-6R treatment depends on the levels of CRP found in patients at baseline. Anti-IL-6R antibodies promote survival of COVID-19 patients presenting initial CRP levels above but not below 15 mg/mL [50]. Based on these results and facing a strong resurgence of the pandemic, the UK regulatory authorities approved the use of anti-IL-6R antibodies to treat critically ill COVID-19 patients. Interestingly, IL-1-receptor (IL-1R) blockade by Anakinra^©^ reduces the expression of IL-6 and of CRP [51,52] and proved superior to Tocilizumab^©^ in promoting COVID-19 survival [53,54]. Thus, IL-1R blockade could be an interesting therapeutic alternative to antibodies targeting IL-6R (Table 2).

**Table 2.** Patients-centred therapeutic strategies against COVID-19.

Treatment	Effect on viral load	Effect on inflammation (CRP)	Effect on severity/mortality
*Immuno-modulation*			
Tocilizumab^®^ anti-IL-6R antibodies^a^	None	↘	↘
Tocilizumab^®^ anti-IL-6R antibodies^b^	None	↘	None
Anakinra^®^ anti-IL-1R antibodies^a^	None	↘↘	↘↘
ACE2-negative umbilical cord MSCs^a^	None	↘↘↘	↘↘
Dexamethasone^a^	None	↘↘	↘↘
Dexamethasone^b^	None	↘↘	↗
*Rebalancing the RAS*			
AT2R agonist	None	↘	↘
ACEI	None	N.T. (potential effect)	↘
ARBs	None	N.T. (potential effect)	↘

Note: AT2R, angiotensin receptor-2; ACEIs, angiotensin converting enzyme inhibitors; ARBs, angiotensin receptor blockers; IL, interleukins; MSCs, mesenchymal stem cells; RAS, renin-angiotensin system; ↘, low reduction; ↘↘, medium reduction; ↘↘↘, strong reduction; ↗, increase.

^a^ Hospitalized patients with severe COVID-19.

^b^ Patients with mild COVID-19.

In support of the potential value of patients-centred strategies exhibiting broad anti-inflammatory effects, a reduction of COVID-19 mortality was also achieved following infusion of ACE2-negative umbilical cord mesenchymal stem cells (MSCs) [55] (Table 2). MSCs display no antiviral properties but general anti-inflammatory capacities [55,56]. In a small pilot study of seven patients after treatment with MSCs, plasma CRP and Tumor Necrosis Factor (TNF)-α levels decreased, and the levels of IL-10 were increased compared to the placebo control group [56]. Concomitantly, MSC transplantation induced the expansion of a CD14^+^CD11c^+^CD11b^mid^ regulatory dendritic cell (DC) population and reduced cytokine-secreting CXCR3-positive CD4 and CD8 T cells, and Natural Killer (NK) cells [56]. Further studies analysing the broad anti-inflammatory effects of MSCs in larger clinical trials to confirm these preliminary observations are required.

Similarly, dexamethasone with its broad anti-inflammatory action, including reduction of CRP levels, was approved against severe COVID-19. Dexamethasone limits mortality rates of severely ill COVID-19 patients receiving mechanical ventilation (Table 2) [57]. However, dexamethasone is detrimental in the early stages of the disease in patients with mild to moderate symptoms not receiving oxygen therapy [57]. This observation is consistent with the hypothesis that the immune response against COVID-19 is divided into two phases, as proposed by Shi et al. [58]. In the first stages of the disease, inflammation potentially promotes a protective antiviral immune reaction linked to type I IFNs production by T-lymphocytes [59]. But in the late stages of the disease, neutrophils producing inflammatory cytokines are damaging for the lungs, provoking respiratory failure and death [58,59]. It has also been proposed that a specific neutrophils subset with immunomodulatory function is responsible for the lymphopenia observed in COVID-19 patients [59]. This emphasizes the specific role of neutrophils in this disease. Similarly, macrophages and DC, able to secrete pro- and anti-inflammatory cytokines, might also play an important role in cytokine storm and lymphopenia [60].

Altogether, patients-centred treatments with broad anti-inflammatory properties have been shown to mitigate COVID-19 severity and mortality of critically ill patients. However, despite the repurposing of these drugs, the COVID-19 death toll remains high [57]. Therefore, additional therapeutic options must be evaluated.

#### Potential benefits of rebalancing the RAS

Older age is a major risk factor of severity of symptoms and death in patients infected by SARS-CoV-2 [36–38,48]. It has been proposed that aging leads to chronic activation of the RAS classical arm and increases the production of Ang-II [48,61]. In turn, by promoting inflammation and the progression of cell senescence, Ang-II could induce cardiovascular [62], respiratory tract and immune system dysregulation, leading to ARDS as well as heart and kidney injuries [63]. These observations could explain the increased susceptibility to severe symptoms and mortality of COVID-19 of the elderly [61]. Indeed, in a large animal model, it has been shown that imbalance of the RAS reproduces some characteristics of the COVID-19 pathophysiology [64].

Moreover, it has been proposed that modifications in ACE2 function due to SARS-CoV-2 spike protein attachment [15] results in further imbalance of the RAS (reviewed in Ref. [65]). SARS-CoV-2 infection has been shown to increase levels of Ang-II [66]. Elevated Ang-II concentration through activation of its AT1R receptor could be responsible for vasoconstriction, thrombosis, inflammation and ARDS, and results in death of patients with COVID-19 [65–67]. Conversely, SARS-CoV-2 binding to ACE2 induces a decrease in levels of angiotensin-(1–7), which mainly oppose the effects of Ang-II on AT1R [68] by activating the Mas receptor (MasR) [68–70]. Thus, it is hypothesized that counterbalancing the effects of SARS-CoV-2 on the RAS has potential anti-inflammatory, anti-fibrotic, antithrombotic, vasodilatory and cardioprotective effects and could reduce mortality in COVID-19 patients with severe pneumonia [65,71,72]. Interestingly, it has been suggested that the efficacy of corticosteroids including dexamethasone to reduce COVID-19 mortality could partly be attributed to their capacity to rebalance the RAS by increasing the activity of ACE2 [73]. Several clinical trials directly testing the effects of restoration of the RAS balance, to reduce mortality due to COVID-19, are now ongoing [65]. Restoring RAS balance could be achieved through many means, including injections of soluble human recombinant ACE2 [19], AT2R activators [68], AT1R blockers (ARBs) [65] or angiotensin converting enzyme inhibitors (ACEIs) [67]. Accordingly, an agonist of AT2R improves respiratory functions and reduces mortality in COVID-19 patients [74]. Similarly, it has been recently shown that the treatment with ACEIs or ARBs during COVID-19 hospitalization reduces mortality [75,76]. This beneficial effect was greater in hypertensive patients and those taking ARBs [75,76]. We are currently clinically testing the effects of BIO101, a pharmaceutical grade preparation of 20-hydroxyecdysone in COVID-19 patients [77]. 20-Hydroxyecdysone has proved to be beneficial *in vivo* in a mouse model of acute lung injury [78]. Furthermore, BIO101 potentially displays anti-fibrotic, vasodilatory [79] and respiratory and cardioprotective effects associated with restoring RAS balance through MasR activation [65,80]. The results of these clinical trials testing BIO101 and other compounds aiming to rebalance the RAS are eagerly waited for. Hopefully, this therapeutic strategy could improve the clinical management of hospitalized COVID-19 patients with severe symptoms.

## Discussion

At present, massive vaccination campaigns are ongoing aiming to limit the spread of historical SARS-CoV-2 strains and emerging SARS-CoV-2 variants but effective treatments are still needed to cope successfully with COVID-19. The current National Institute of Health (NIH) guidelines recommend to use infusion of neutralizing antibodies and antiviral drugs in the early stages of the disease, followed by the use of corticosteroid associated or not with administration of anti-IL-6R antibodies with severe symptoms. Despite the vast effort from the medical and scientific community to provide the best supportive care possible to hospitalized patients, the mortality associated with COVID-19 pandemic remains important. Therefore, new therapeutic options limiting the severity and mortality of hospitalized COVID-19 patients are still required. Based on our literature review, we propose that rather than virus-centred strategies, patients-centred strategies hold more promise for the development of future curative treatments able to reduce COVID-19 mortality of patients hospitalized with severe symptoms. Among them, drugs rebalancing the RAS, modulating multiple physiological processes, disturbed during normal aging and following SARS-CoV-2 infection could prove effective therapies against COVID-19. We are currently clinically testing BIO101 that could restore RAS balance in COVID-19 patients. If successful, BIO101 and other drugs aiming to restore RAS balance could find their place in the therapeutic landscape against COVID-19 and hopefully could help us return to “normal” life.
